# Prevention survey in Bavaria – an approach to prevention reporting at the federal state level?

**DOI:** 10.17886/RKI-GBE-2017-095

**Published:** 2017-08-30

**Authors:** Veronika Reisig, Joseph Kuhn

**Affiliations:** Bavarian Health and Food Safety Authority, Department Health Reporting, Epidemiology, Social Medicine

## Abstract

The Preventive Health Care Act stipulates the production of a national prevention report with the option of contributions by the federal states. Bavaria is currently establishing a system of prevention reporting. This effort aims to provide information on health related aspects relevant to prevention and health promotion and, through intervention reporting, deliver also a picture of current prevention measures. An online survey took stock of the measures of primary prevention and health promotion. This provided an overview of the Bavarian prevention landscape with respect to activities as well as aspects related to the structure and quality and allowed to derive recommendations for further development. Moreover, this article highlights the limitations of such a survey.

## Background

Issues of prevention have always been an integral part of health reporting. Currently, the Preventive Health Care Act, which was adopted in 2015, foresees the production of a prevention report with a potential contribution by the federal states. In the justification of the law the role played by health reports and health maps for the regional co-ordination of prevention measures and policies is emphasised. For prevention reporting in the stricter sense, however, hardly any conceptual groundwork has been undertaken so far [1].

Against the backdrop of the Bavarian Prevention Plan, Bavaria is establishing a prevention reporting system to support Bavarian prevention policies, as well as regional initiatives, and contribute towards national level reporting [2].

## The concept for prevention reporting in Bavaria

Prevention reporting aims to provide and interpret data on population health in terms of prevention needs, potentials and outcomes, as well as to describe prevention activities and structures. Bavaria’s concept for prevention reporting is organised in modules which will comprise indicators of health related aspects relevant to prevention and/or health promotion as well as provide information on prevention measures in the form of intervention reporting.

## Surveying prevention in Bavaria

**Figure 1:**
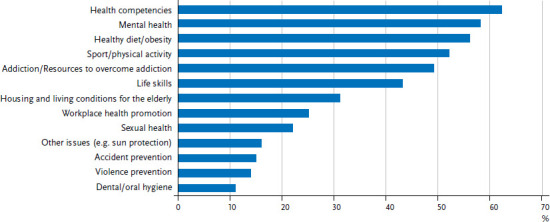
Most frequently mentioned prevention topics by stakeholders in Bavaria (in % of stakeholders, n=135) Source: Institut für Gesundheits- und Sozialforschung [4]

In 2014/2015 Bavaria took stock of the activities and structures of primary prevention and health promotion. A broad spectrum of stakeholders (around 600 organisations) was surveyed online. Moreover, nine experts were interviewed to assess civic and corporate commitment. 135 stakeholders (around 23%) eventually took part in the online survey, among them many of the large players in the field of prevention such as, for example, four of Bavaria’s five largest statutory health insurance funds and three of the six largest charities [3]. The survey provides an overview of the Bavarian prevention landscape with regard to target groups, key issues and approaches, as well as aspects of quality and structure (see, as an example, figure 1). These results provided a basis to derive recommendations on strategic as well as on quality related issues.

The survey is limited by the nature of its object of inquiry, primary prevention and health promotion, which is a field that is difficult to define and highly complex, as well as by the heterogeneity of the stakeholders, which does not allow to completely capture them all. To a certain degree, this blurs the overall picture regarding quantitative aspects and the relation between individual aspects. The study design does not allow conclusions to be drawn on the effects of health promotion and primary prevention on the health of the population.

## Discussion and outlook

The survey provides added value by giving an indicative overview of the stakeholders, activities, topics and aspects of quality assurance in prevention in Bavaria, in particular with regard to the Bavarian Prevention Plan. Within this context, the survey can contribute towards a discussion of the current situation in Bavaria and perspectives for future developments. The question of whether the efforts such a survey requires are justified, or whether, in future, sectoral or regional surveys should be given priority, remains open.
